# Supplementary material for: Comparative Analysis of Floral Transcriptomes in Gossypium hirsutum (Malvaceae)
Source: Plants (Basel). 2025 Feb 7;14(4):502. doi: 10.3390/plants14040502 (PMC11859044; doi:10.3390/plants14040502)
Supplement: Supplementary file 1 [file plants-14-00502-s001.zip › Supplementary_Figures.zip!/Supplementary_Figures.pdf]

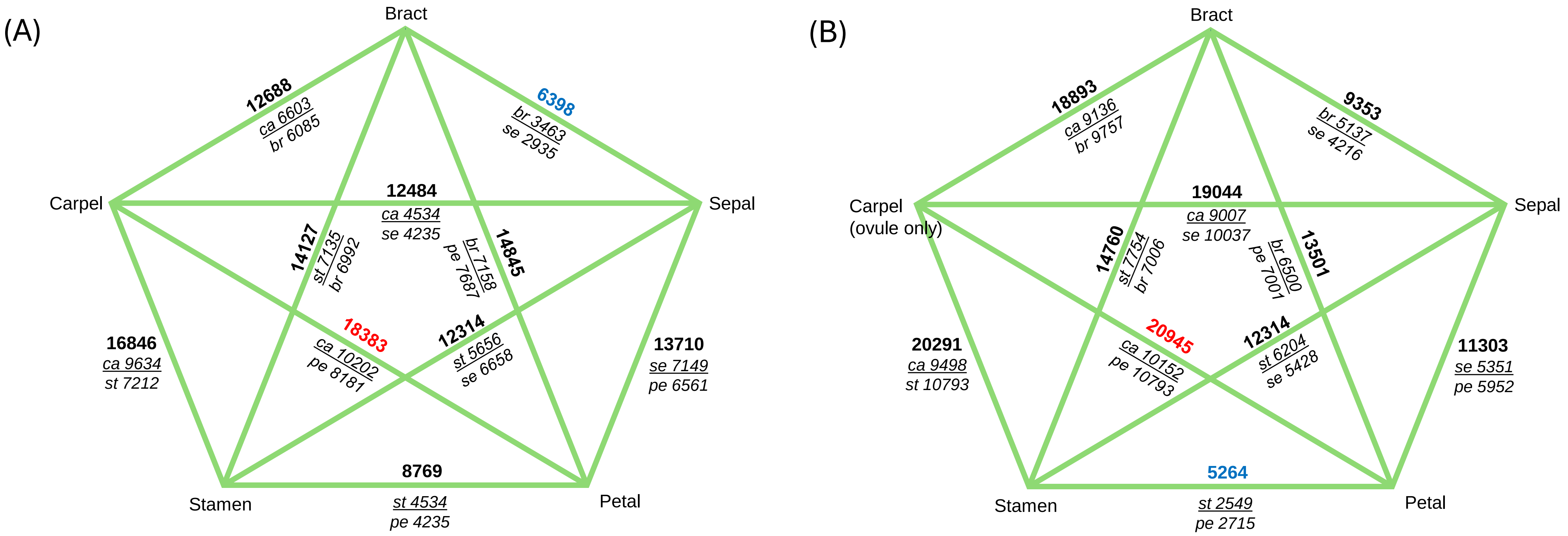

Figure S1: Number of differentially expressed (DE) genes between organs of (A) TX2094 and (B) TM-1. The highest and lowest number of DE genes were shown in red and blue text, respectively. The organ-specific breakdown was shown below the total DE number. For example, in the comparison between carpel and bract, a total of 12688 DE genes were identified. Of these, 6605 and 6085 genes were more abundant in carpel and bract, respectively. br: bract, se: sepal, pe: petal, st: stamen, ca: carpel.

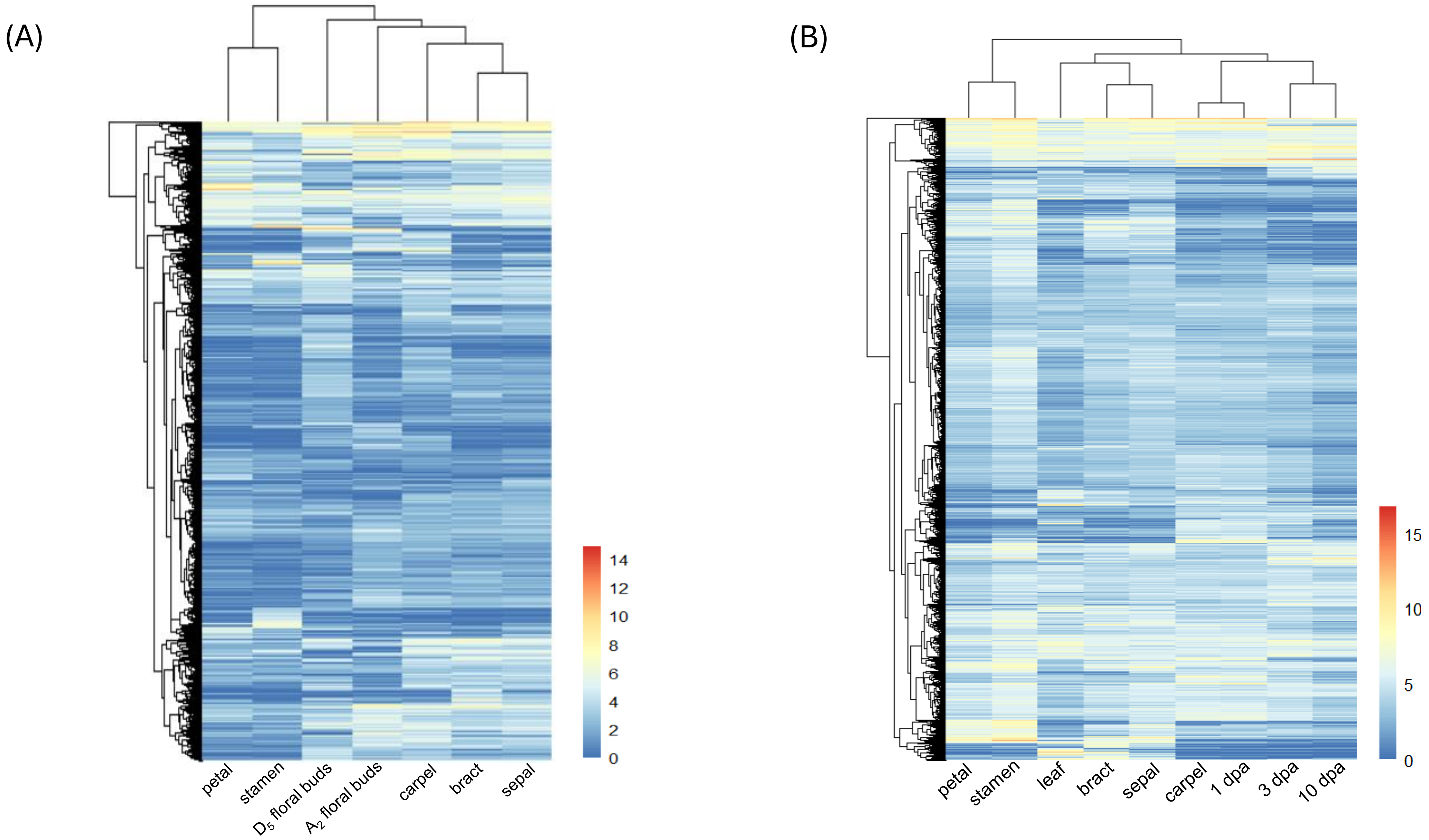

Figure S2: Hierarchical clustering of expressions of transcripts expressed in *G. hirsutum* (A) TX2094 and (B) TM-1 organs and the floral buds of *G. arboreum* (A_2) and *G. raimondii* (D_5).

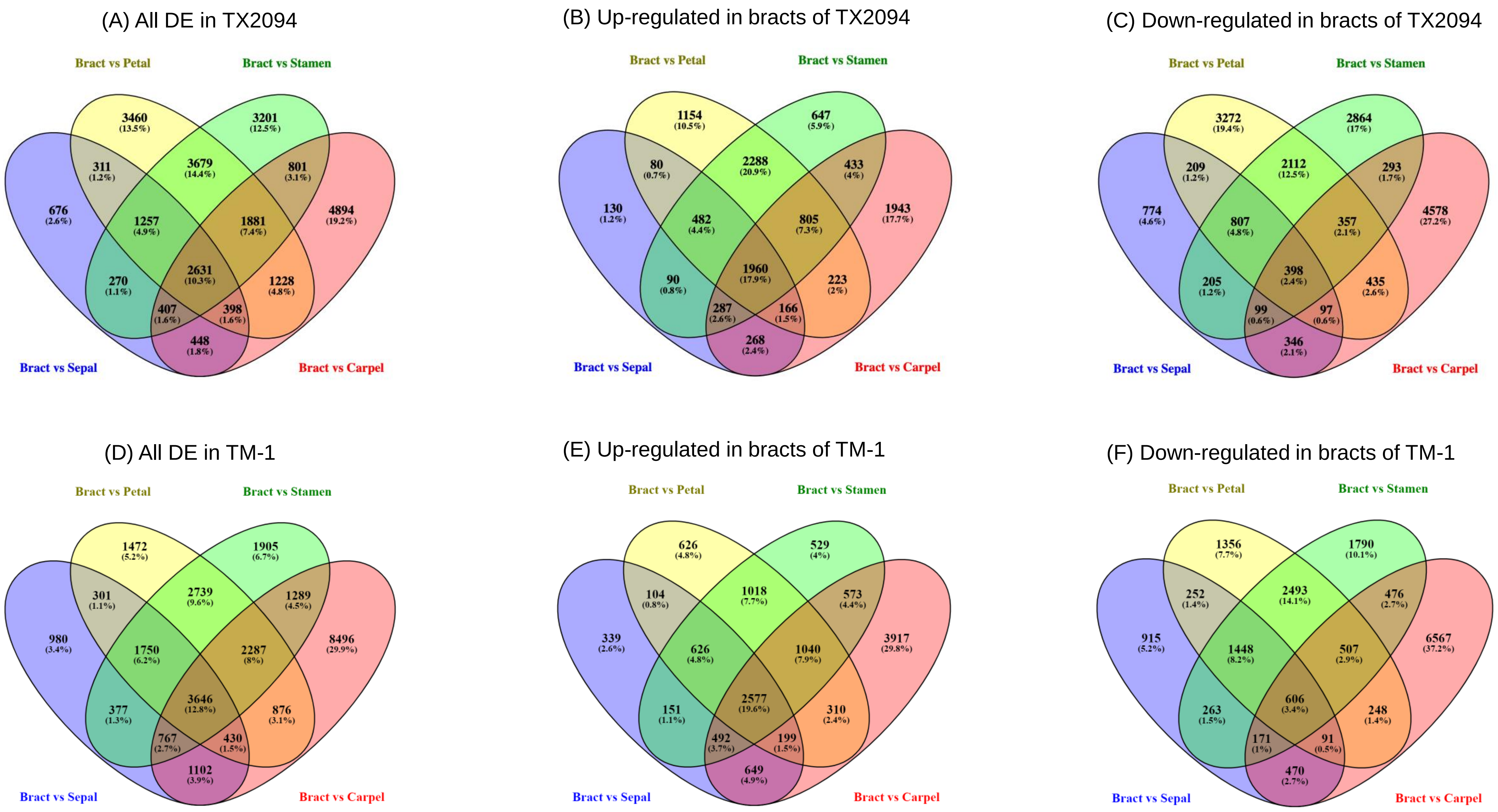

Figure S3: Comparison of differentially expressed (DE) transcripts between organ types in TX2094 and TM-1.

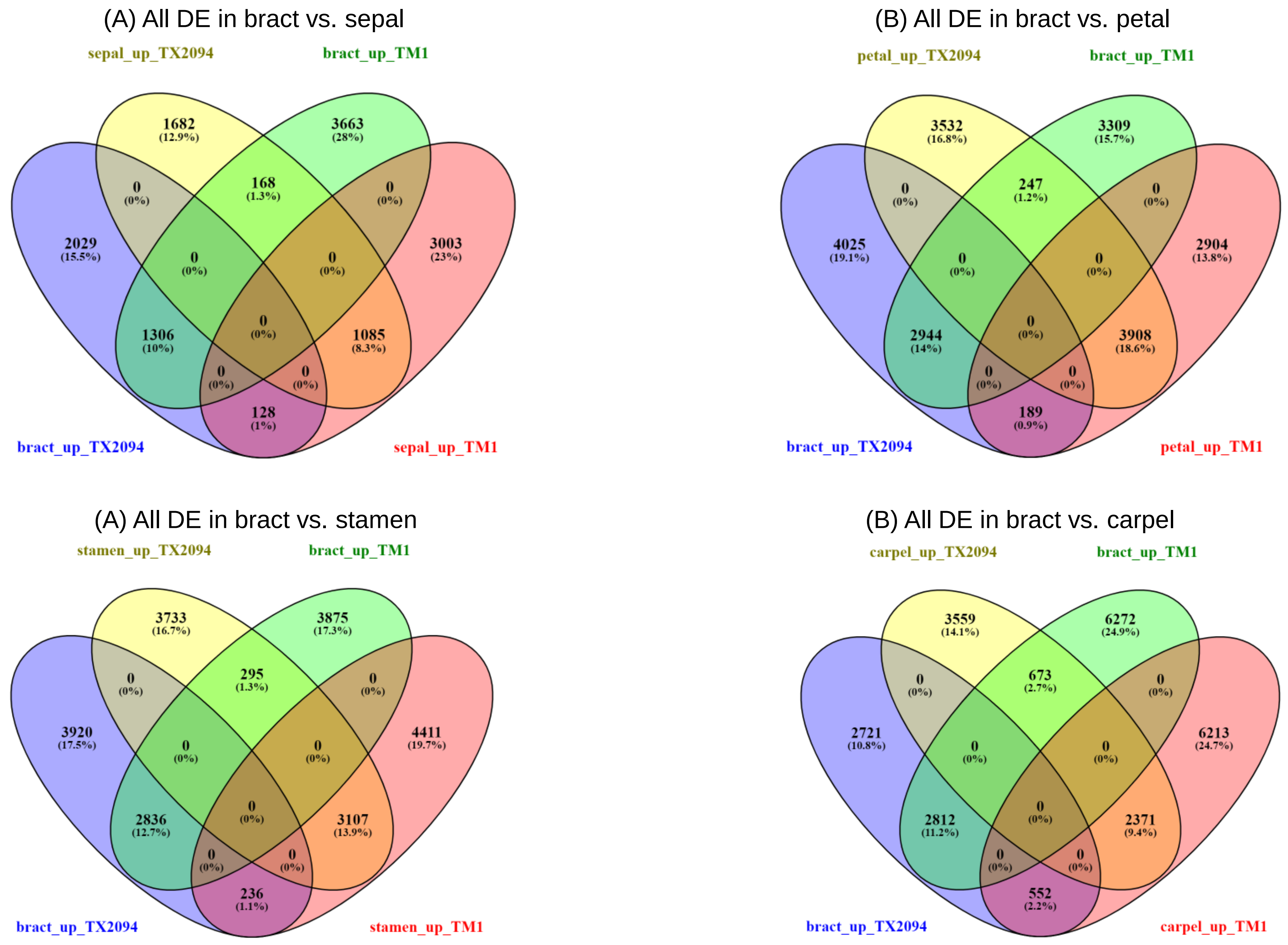

Figure S4: Comparison of differentially expressed (DE) transcripts between TX2094 and TM-1.

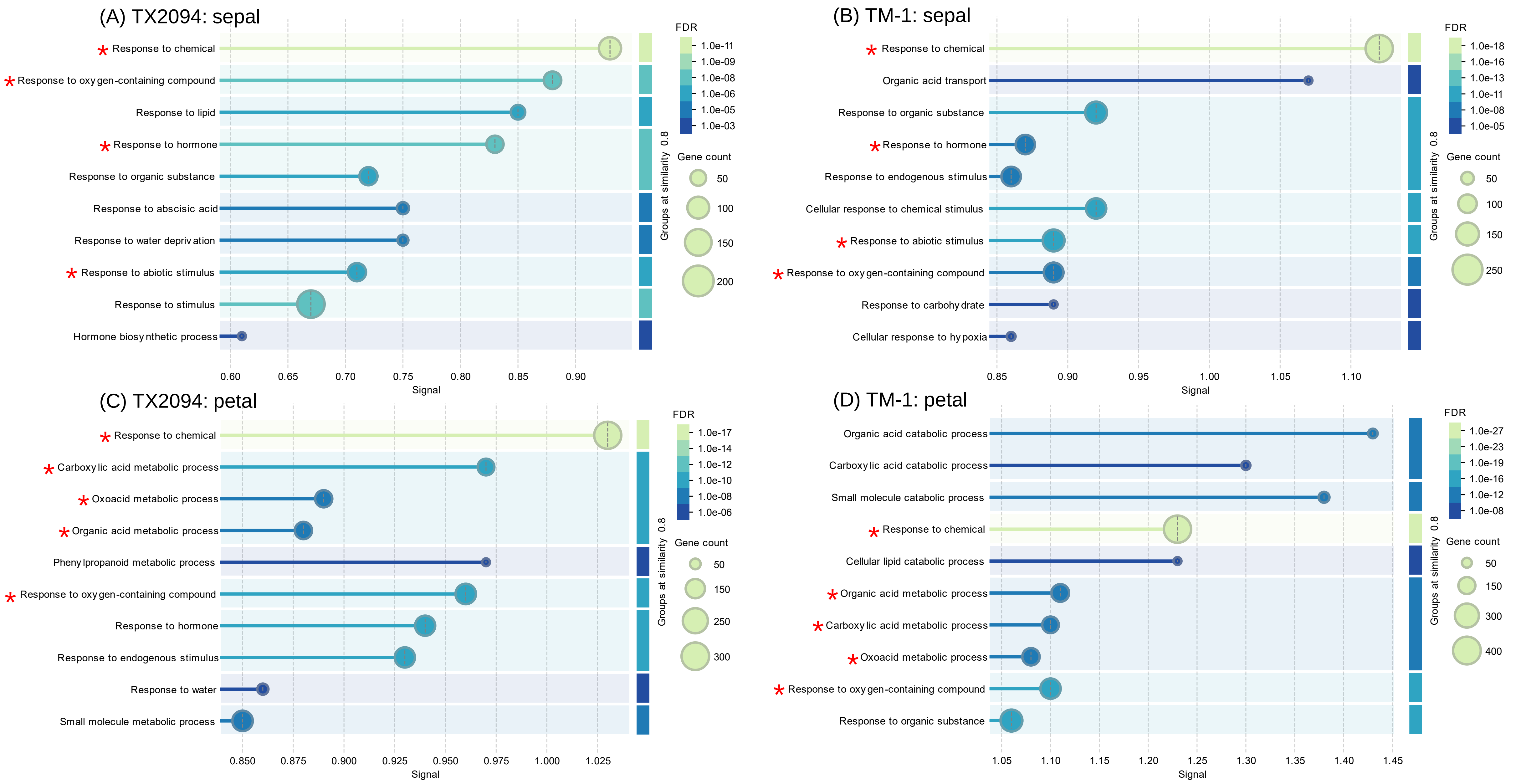

Figure S5: The results of biological process (BP) enrichment tests in each organ of TX2094 and TM-1. * shows common BP in both accessions.

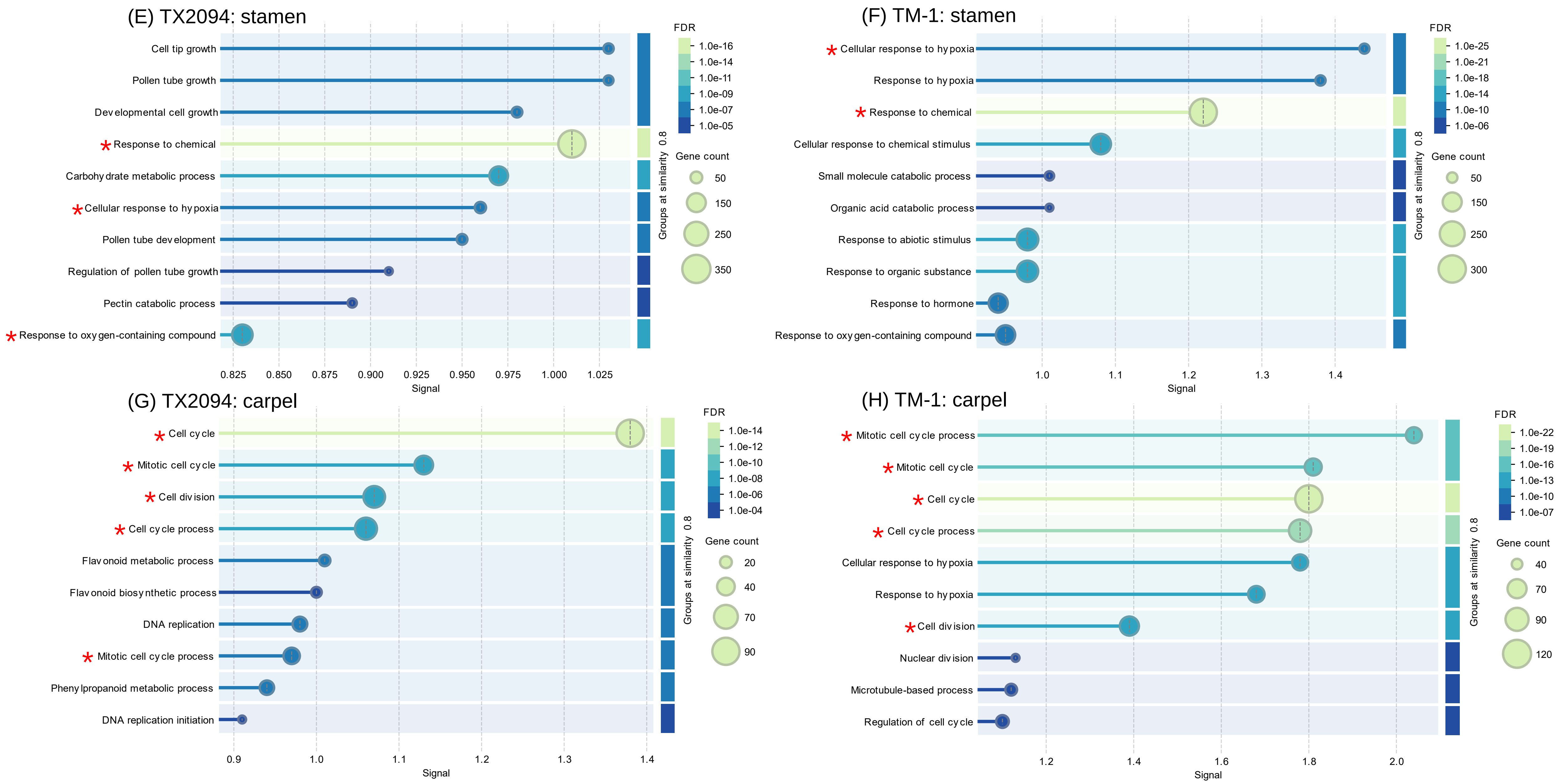

Figure S5: Continued.

(A) STRING analysis of TFs differentially expressed in sepals of TX2094 (left) and TM-1 (right)

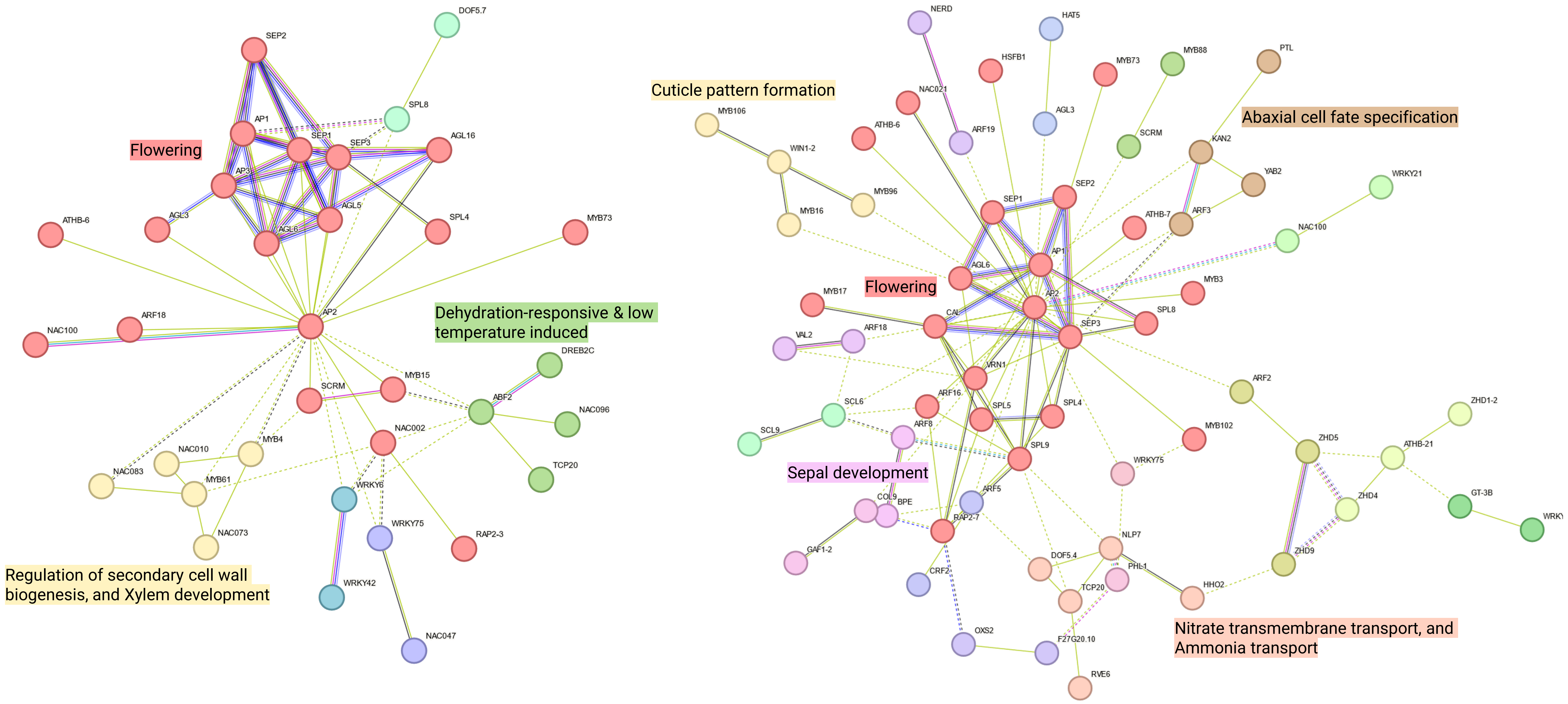

Figure S6: Clusters from STRING analysis of transcription factors differentially expressed in each floral organ relative to bracts.

(B) STRING analysis of TFs differentially expressed in petals of TX2094 (left) and TM-1 (right)

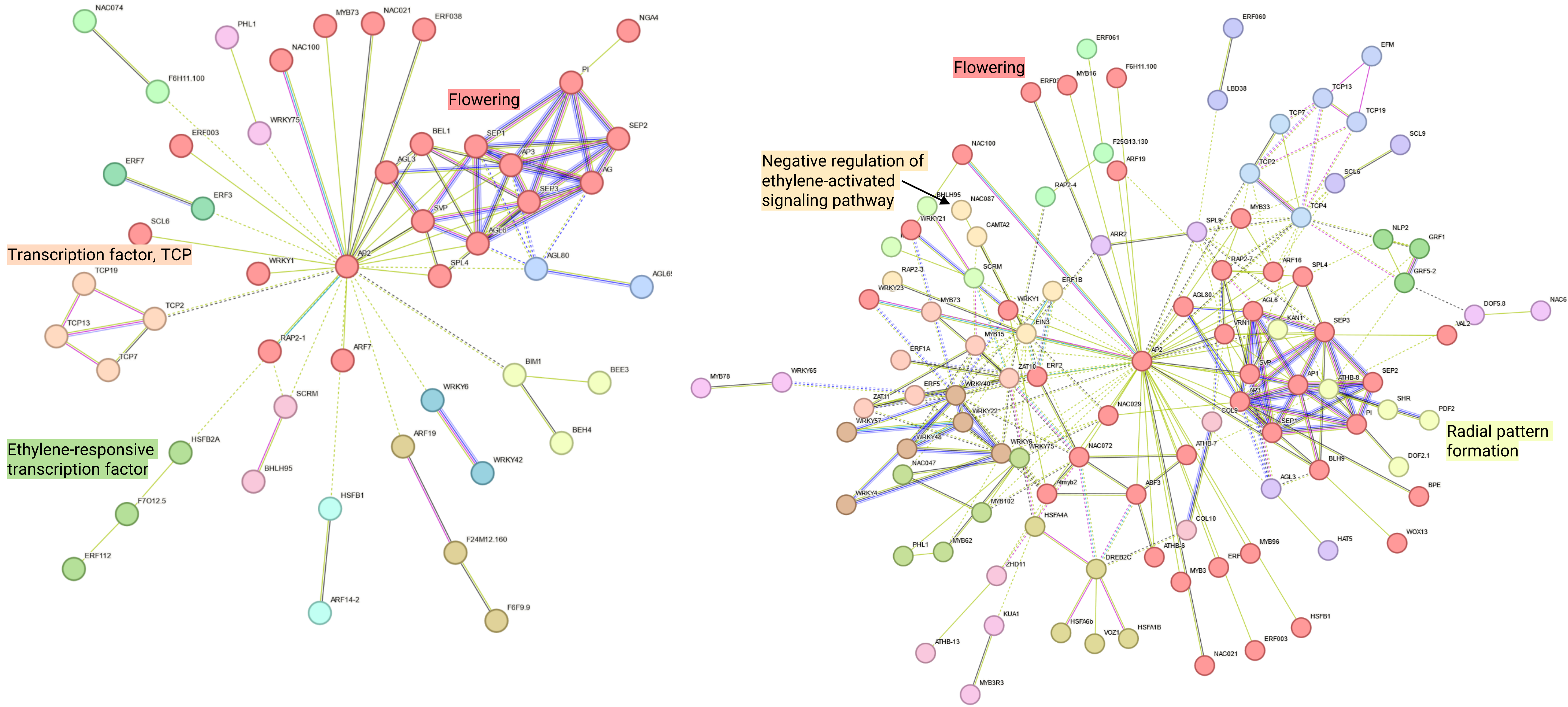

Figure S6: Continued.

(C) STRING analysis of TFs differentially expressed in stamens of TX2094 (left) and TM-1 (right)

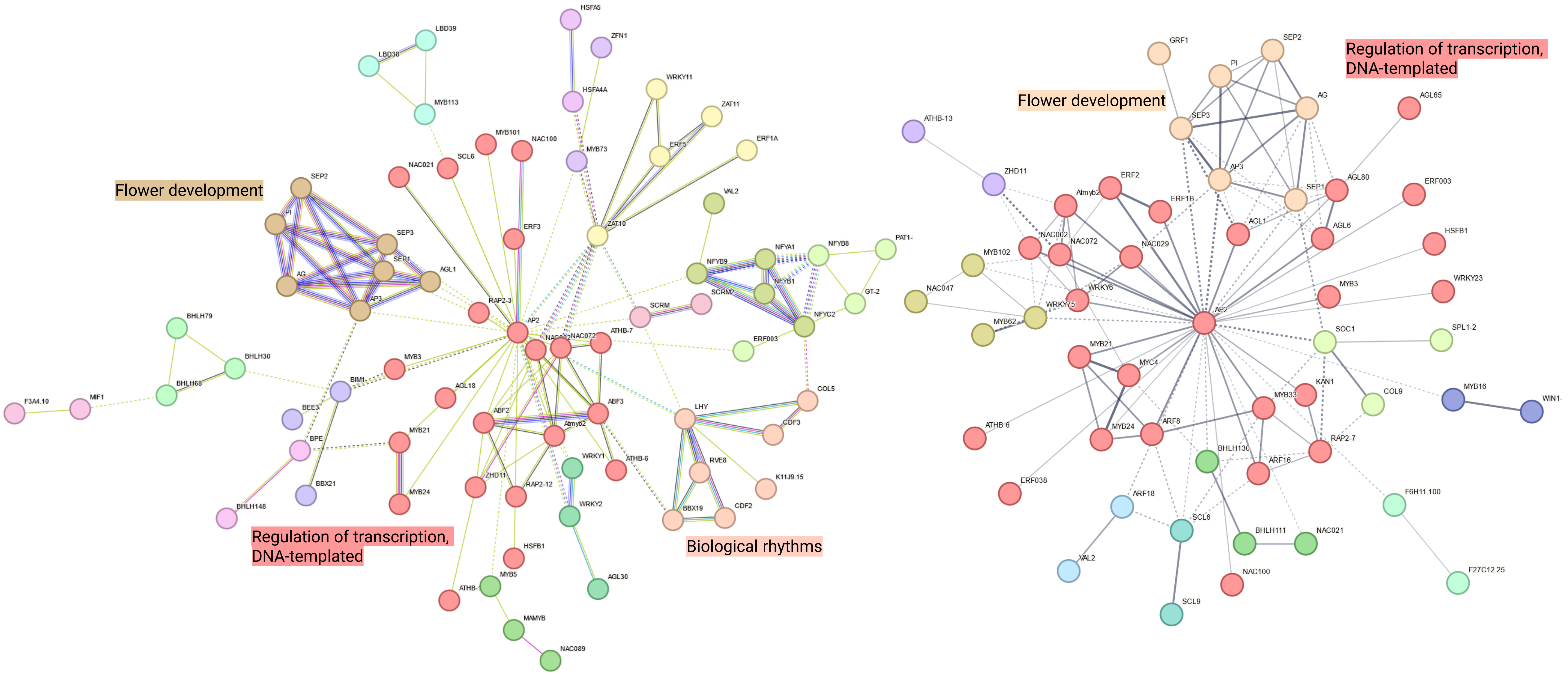

Figure S6: Continued.

(D) STRING analysis of TFs differentially expressed in carpels of TX2094 (left) and TM-1 (right)

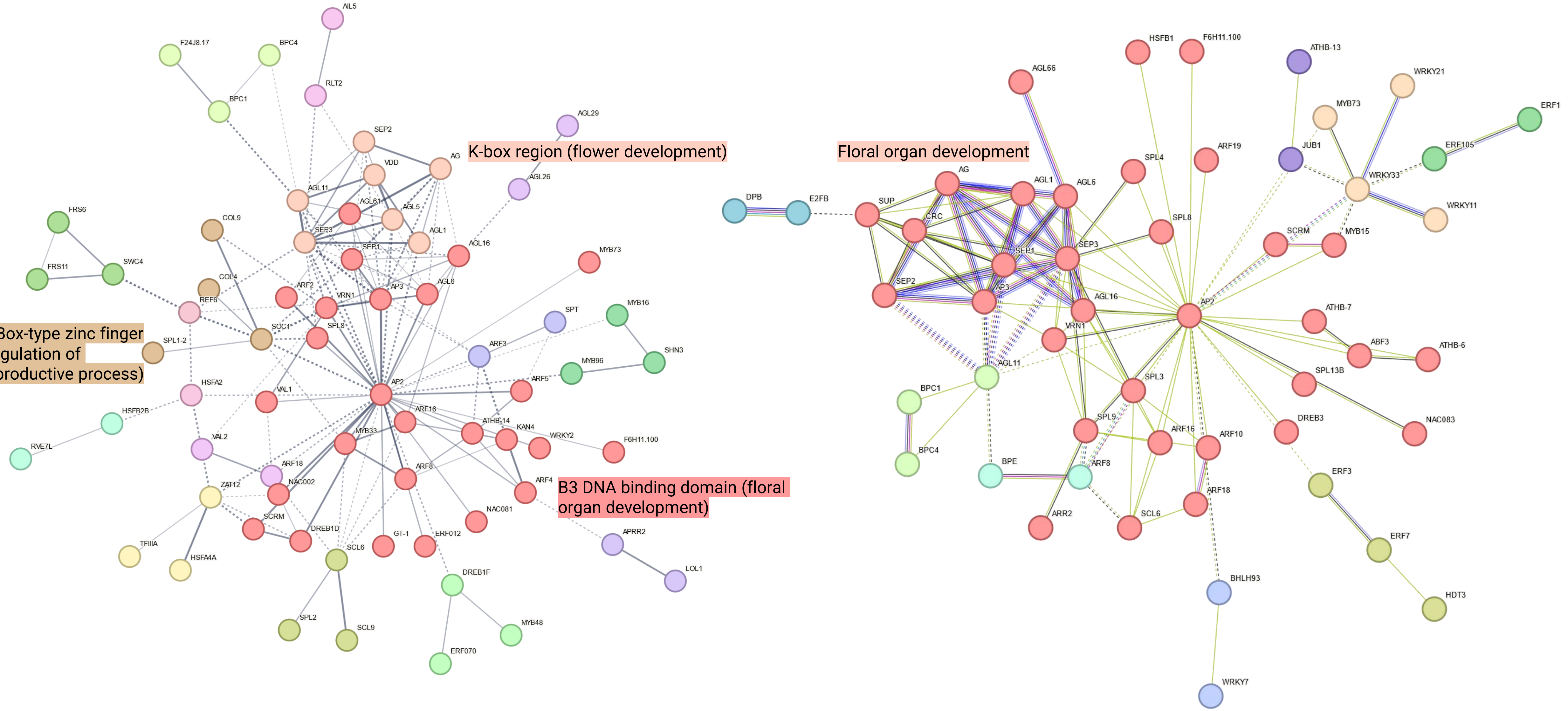

Figure S6: Continued.

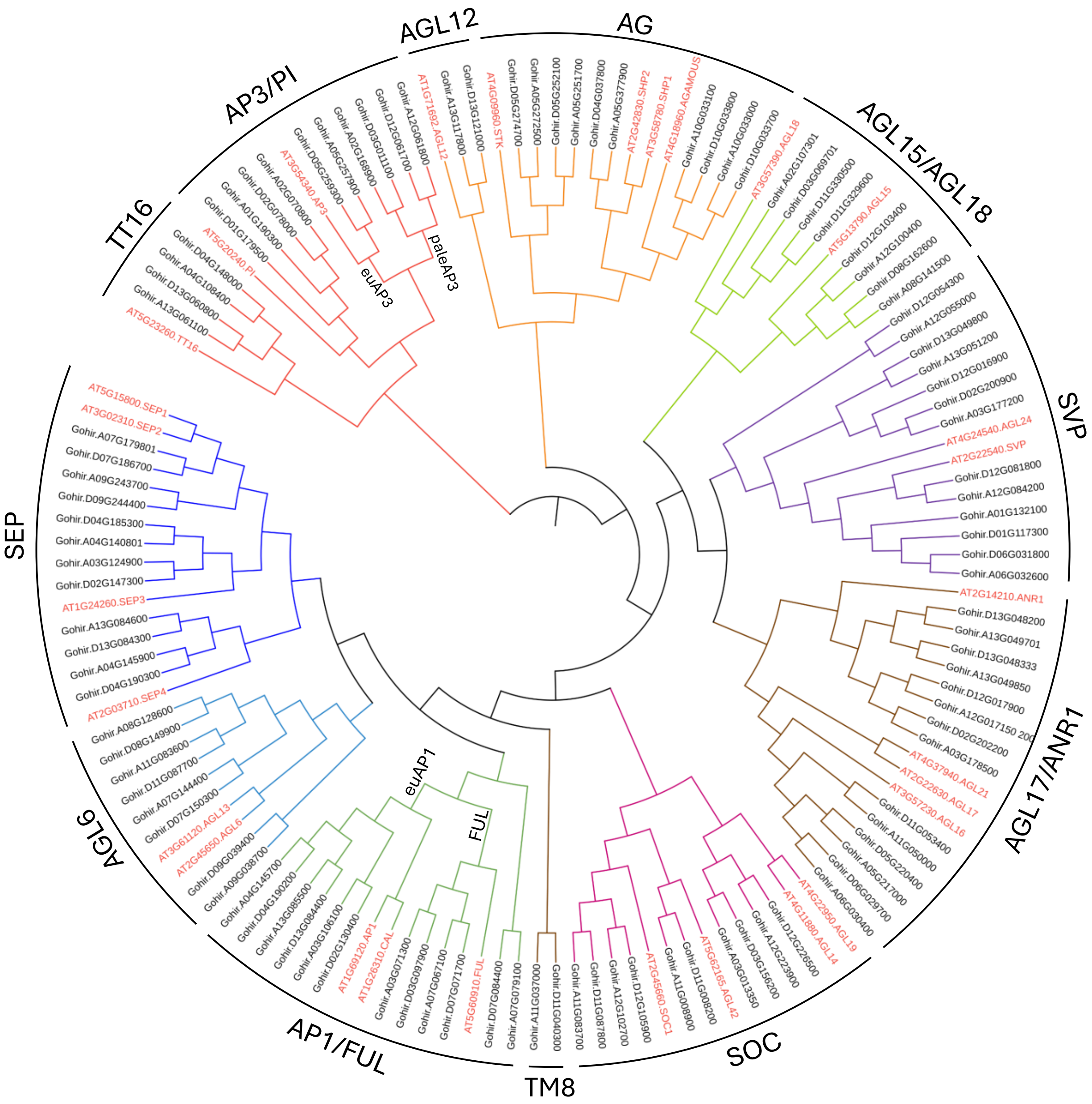

Figure S7. Maximum likelihood tree of *G. hirsutum* MIKC-type MADS-box genes. *Arabidopsis* homologs were shown in red.

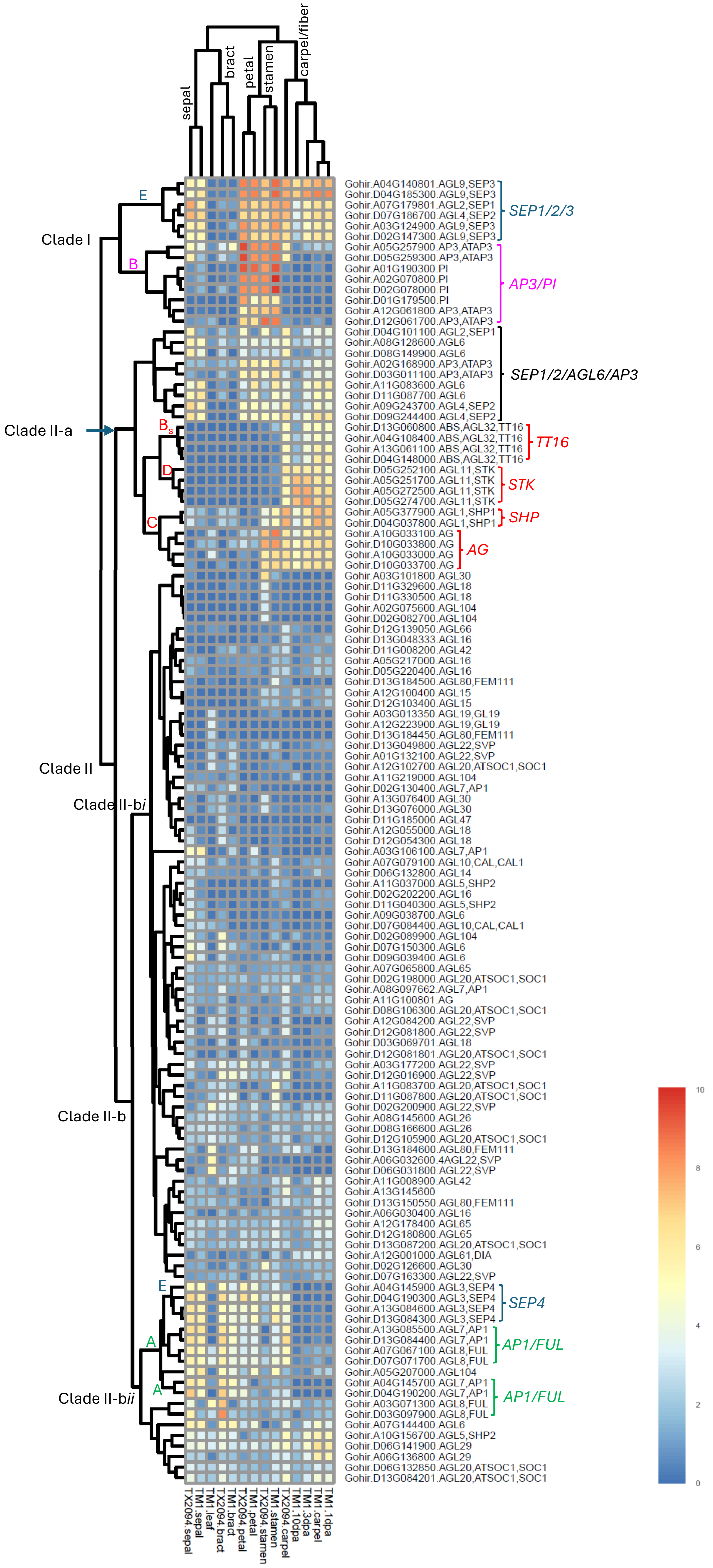

Figure S8: Hierarchical clustering of expressions of *G. hirsutum* MADS-box genes. The color scale bar in the right, bottom corner shows high-expression (red) and no expression (0 TPM; blue). The subclade name was given next to *G. hirsutum* ID

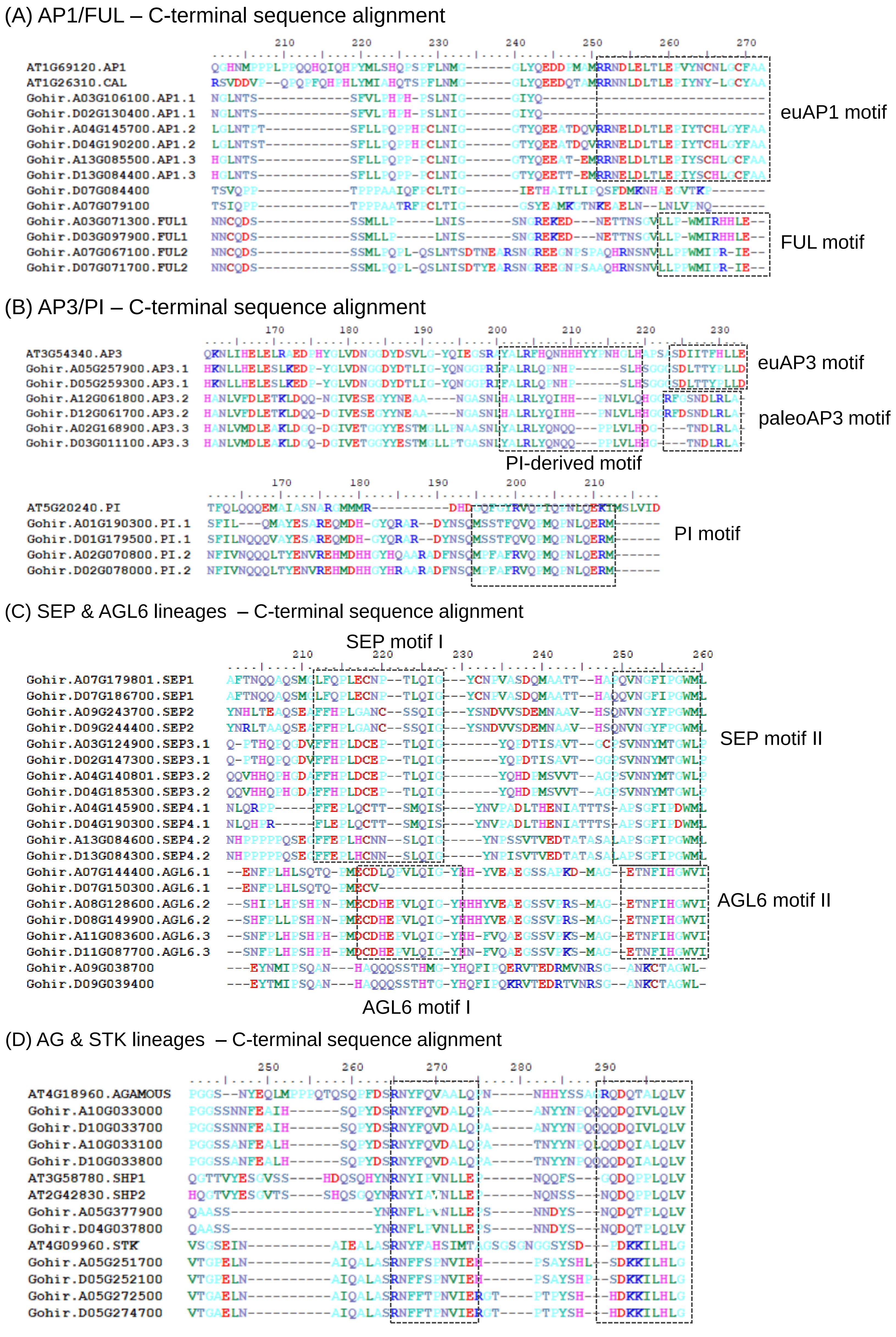

Figure S9: Motif comparison of MIKC-type MADS-box genes

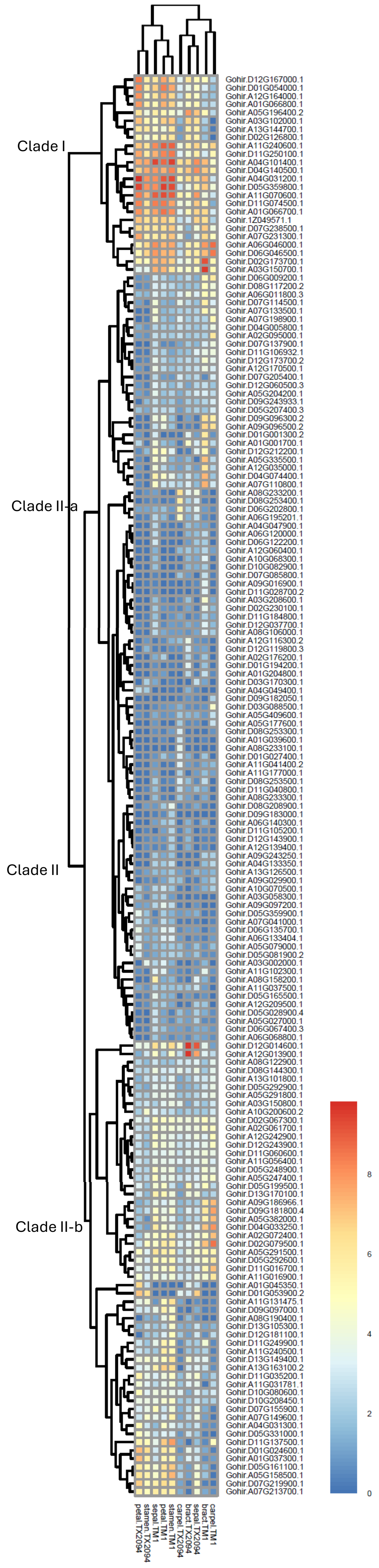

Figure S10: Hierarchical clustering of expressions of *G. hirsutum NAC* genes. The color scale bar in the right, bottom corner shows high-expression (red) and no expression (0 TPM; blue). The subclade name was given next to *G. hirsutum* ID

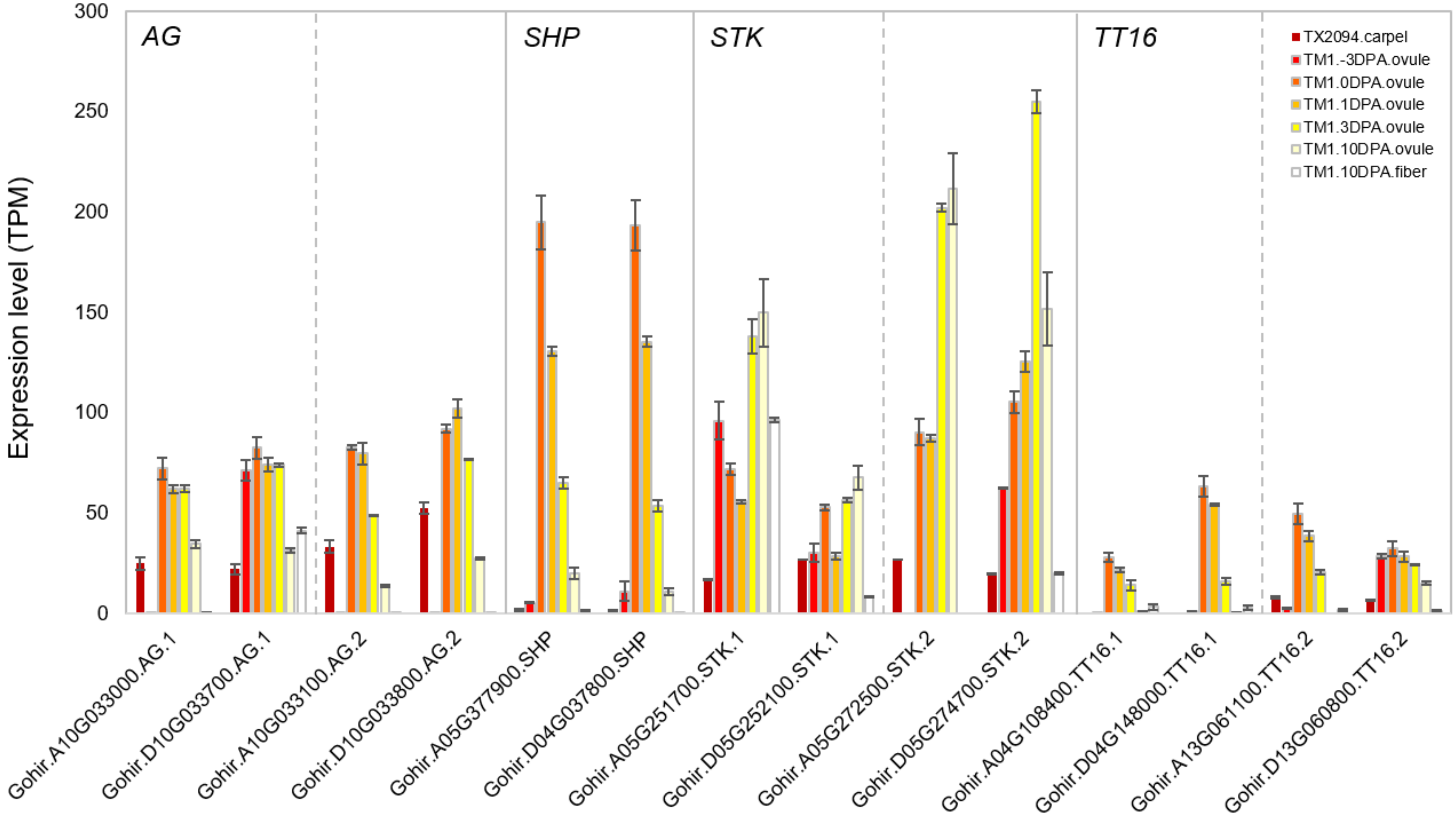

Figure S11: Expression patterns of genes related to carpel (ovule) development in TX2094 and TM-1.

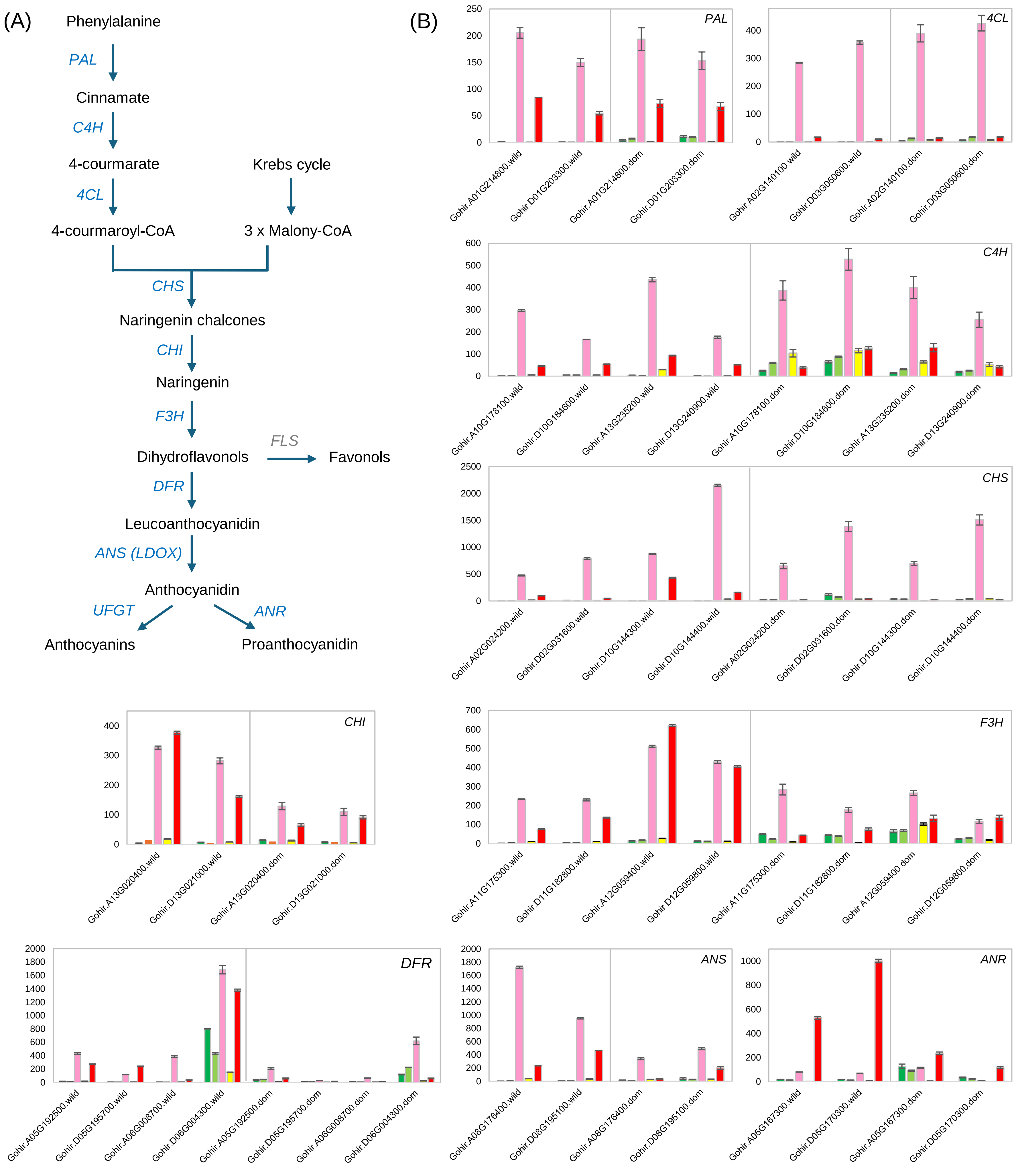

Figure S12: Expression of genes related to flavonoid biosynthetic pathway (A) and their expression levels (B) in wild and domesticated cotton. wild = TX2094, dom = TM-1, *PAL*, phenylalanine ammonium lyase; *C4H*, cinnamate-4-hydroxylase; *4CL*, 4-coumaroyl-CoA synthase; *CHI*, chalcone isomerase; *F3H*, flavonol 3-hydroxylase; *FLS*, flavonol synthase; *DFR*, dihydroflavonol-4-reductase; *ANS*, anthocyanin synthase, *LDOX*, leucoanthocyanidin dioxygenase; *ANR*, anthocyanidin reductase. In the bar chart, each organ is represented by a different color: bracts = green, sepals = light green, petals = pink, stamens = yellow, and carpels = red.

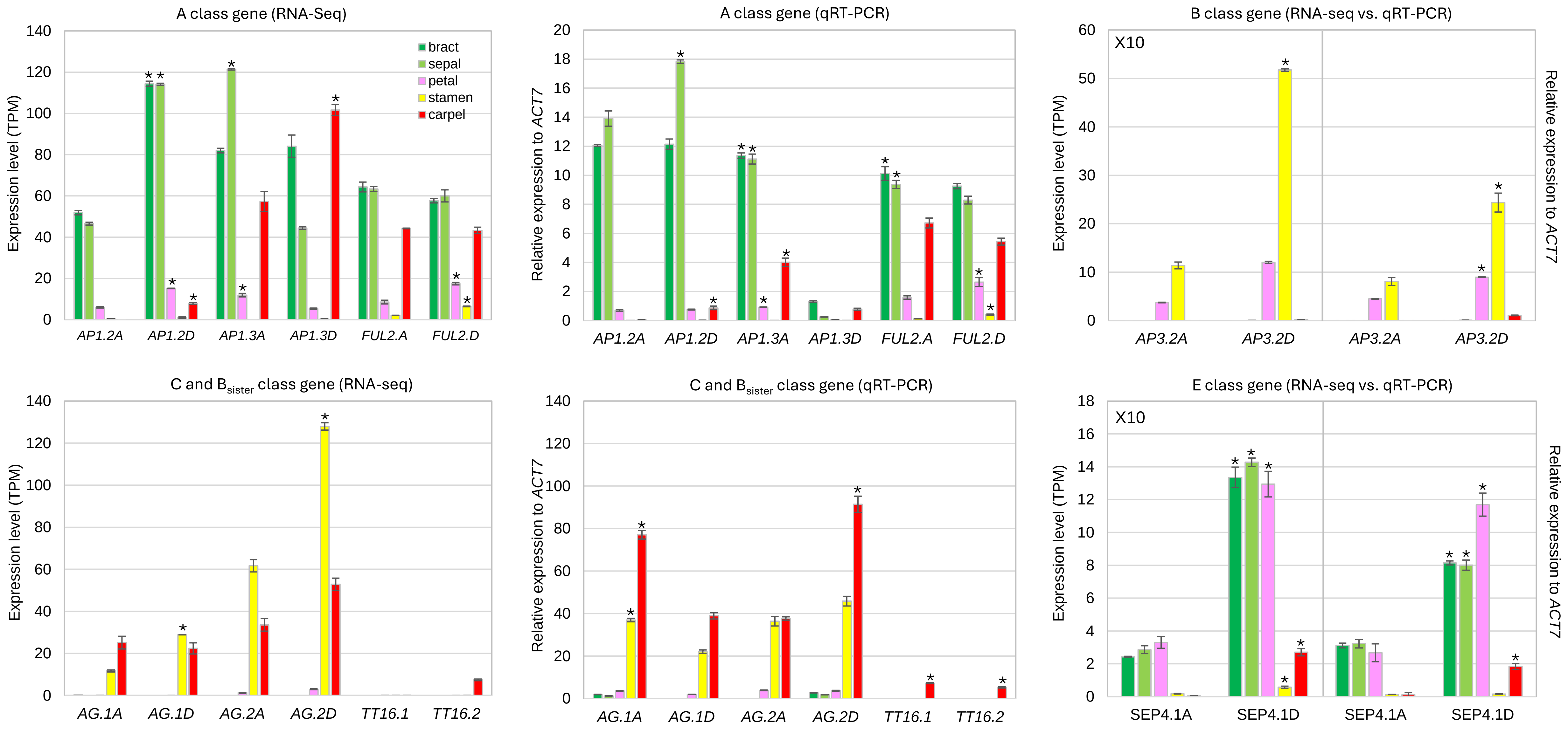

Figure S13: The validation of gene expression level in RNA-seq using qRT-PCR. The expression levels for *AP3* and *SEP4* were reduced by 10 times.
